# *In vitro* antioxidant activity of pet ether extract of black pepper

**DOI:** 10.4103/0253-7613.43160

**Published:** 2008-08

**Authors:** Ramnik Singh, Narinder Singh, B.S. Saini, Harwinder Singh Rao

**Affiliations:** Sri Sai College of Pharmacy, Badhani, Pathankot, Gurdaspur, India; 1 Department of Pharmacology, SGRD Medical Institute, Amritsar, India; 2 Institute of Pharmaceutical Sciences and Engineering, Amritsar, India

**Keywords:** Lipid peroxidation, phenolic compounds, *Piper nigrum*

## Abstract

**Objective:**

To investigate the *in vitro* antioxidant activity of different fractions (R1, R2 and R3) obtained from pet ether extract of black pepper fruits (*Piper nigrum* Linn.)

**Materials and Methods:**

The fractions R1, R2 and R3 were eluted from pet ether and ethyl acetate in the ratio of 6:4, 5:5 and 4:6, respectively.

1,1-Diphenyl-2-picryl-hydrazyl (DPPH) radical, superoxide anion radical, nitric oxide radical, and hydroxyl radical scavenging assays were carried out to evaluate the antioxidant potential of the extract.

**Results:**

The free radical scavenging activity of the different fractions of pet ether extract of *P. nigrum* (PEPN) increased in a concentration dependent manner. The R3 and R2 fraction of PEPN in 500 *µ*g/ml inhibited the peroxidation of a linoleic acid emulsion by 60.48±3.33% and 58.89±2.51%, respectively. In DPPH free radical scavenging assay, the activity of R3 and R2 were found to be almost similar. The R3 (100*µ*g/ml) fraction of PEPN inhibited 55.68±4.48% nitric oxide radicals generated from sodium nitroprusside, whereas curcumin in the same concentration inhibited 84.27±4.12%. Moreover, PEPN scavenged the superoxide radical generated by the Xanthine/Xanthine oxidase system. The fraction R2 and R3 in the doses of 1000*µ*g/ml inhibited 61.04±5.11% and 63.56±4.17%, respectively. The hydroxyl radical was generated by Fenton's reaction. The amounts of total phenolic compounds were determined and 56.98 *µ*g pyrocatechol phenol equivalents were detected in one mg of R3.

**Conclusions:**

*P. nigrum* could be considered as a potential source of natural antioxidant.

## Introduction

The importance of the reactive oxygen species (ROS) has attracted increasing attention over the last decade. Reactive oxygen species includes free radicals such as superoxide anion radicals (O_2_^−•^), hydroxyl radicals (OH^−^), non free radicals such as H_2_O_2_ and singlet oxygen (^1^O_2_), along with various forms of activated oxygen. They are involved in various physicochemical processes and diseases such as aging,[1] cancer[2] and activity atherosclerosis.[3] Several studies have reported that plants have potent antioxidant and represent an important source of natural antioxidants.[4–6] Butyl hydroxyl anisol (BHA), and butyl hydroxyl toluene (BHT) are the most commonly used antioxidants, but both are suspected to cause liver damage.[7–8]

Black pepper (*Piper nigrum* Linn.), the king of spice, is one of the oldest and most popular spices in the world. It belongs to the family Piperaceae and is used in many Asian countries as a stimulant in the treatment of colic, rheumatism, headache, diarrhoea, dysentery, cholera, menstrual pain, removing excessive gas from gastrointestinal tract and increasing flow of urine. It is used in folk medicine for stomach disorders, digestive problems, neuralgia and scabies. Its active constituent, piperine, has been reported to reduce liver damage in rats.[9] The methanolic extract of *P. nigrum* fruits has hepatoprotective and antioxidant effects in rats.[10] The purpose of the present study was to evaluate the *in vitro* antioxidant activity of different fractions obtained from pet ether extract of *Piper nigrum* (PEPN).

## Materials and Methods

### Plant materials and extraction

*Piper nigrum* Linn. fruits were collected in the month of March 2003, from Kottayam, Kerala, India. The plant materials were identified and authenticated in the Herbarium, Botany Department, Guru Nanak Dev University, Amritsar, Punjab, India. A voucher specimen (B-05) of the collected plant material was also deposited in the Department of Pharmacy, Government Institute of Pharmaceutical Sciences & Engineering, Amritsar, for future reference. The collected materials were washed thoroughly in water, air dried for a week at 35-40°C and pulverized in electric grinder. The powder thus obtained was extracted in pet ether. The extract was then again made to powder, by using a rotary evaporator under reduced pressure. This powder was further subjected to silica gel (#60-120, BDH) column chromatogram and fraction R1 (60.7%), R2 (29.1%) and R3 (64.2%) were eluted from the mixture of pet ether and ethyl acetate in the ratio of 6:4, 5:5 and 4:6 respectively.

### Chemicals

Ammonium thiocyanate was purchased from Merck, Darmstadt, Germany. Ferrous chloride, ferric chloride (FeCl_2_), 1, 1-diphenyl-2-picryl-hydrazyl (DPPH), EDTA, butylated hydroxy toluene (BHT), butylated hydroxy anisole (BHA), α-tocopherol, ascorbic acid, catechin, pyrocatechol, curcumin, nitroblue tetrazolium (NBT), thiobarbituric acid, 2-deoxy-2-ribose, and trichloroacetic acid were purchased from Sigma, St. Louis, USA. All other chemicals and reagents were of analytical grade.

### Determination of total antioxidant activity

The antioxidant activity of different fractions (R1, R2 and R3) obtained from PEPN was determined using the thiocyanate method.[11] Various concentrations (50, 100, 250, and 500 *µ*g/ml) of different fractions of PEPN were prepared in methanol and added to a linoleic acid emulsion (2.5 ml, 40 mM, pH 7.0) and phosphate buffer (2 ml, 40 mM, pH 7.0). The linoleic acid emulsion was prepared by mixing 0.2804 g linoleic acid with 0.2804 g Tween-20 as emulsifier in 50 ml 40 mM phosphate buffer. The mixture was then homogenized. The final volume was adjusted to 5 ml with 40 mM phosphate buffer, and pH 7.0. The mixed samples were then incubated at 37°C in a glass flask for 60 h, to accelerate the oxidation process.[12] One milliliter of the incubated sample was removed at 12hour interval and 0.1 ml 20 mM FeCl_2_ and 0.1 ml 30% ammonium thiocyanate was added. The absorbance of this was measured at 500 nm, using a spectrophotometer (DU640i, Beckman). Alpha Tocopherol was used as a reference compound. To eliminate the solvent effect, the control sample, which contained the same amount of solvent added to the linoleic acid emulsion in the test sample and the reference compound, was used. All data reported are the average of triplicate analyses. Percent inhibition of lipid peroxide generation was calculated using following formula.

(1) $$\text{\% Inhibition}=\frac{\text{Control absorbance}-\text{Test absorbance}}{\text{Control absorbance}}$$

### DPPH free radical scavenging activity of PEPN

The free radical scavenging activity of the different fractions of PEPN was measured using DPPH, employing the method of Blois.[13] One ml of each fraction of PEPN and the reference compound in various concentrations (50, 100, 150, 200 & 250 *µ*g/ml) was added to one ml of 0.1 mM solution of DPPH in methanol. After 30 minutes, absorbance was measured at 517 nm, using a spectrophotometer (DU640i, Beckman). A 0.01mM solution of DPPH in methanol was used as control, whereas BHA was used as a reference material. All tests were performed in triplicate. Percent inhibition was calculated using equation 1.

### Nitric oxide radical scavenging activity

Nitric oxide generated from sodium nitroprusside in aqueous solution at physiological pH interacted with oxygen to produce nitrite ions, which were measured using the Griess reaction.[14] Three ml of 10 mM sodium nitroprusside in phosphate buffer was added to two ml of each fraction of PEPN and the reference compound in different concentrations (20, 40, 60, 80, and 100 *µ*g/ml). The resulting solutions were then incubated at 25°C for 60 minutes. A similar procedure was repeated with methanol as a blank, which served as control. To 5 ml of the incubated sample, 5 ml Griess reagent (1% sulfanilamide, 0.1% naphthylethylene diamine dihydrochloride in 2% H_3_ PO_4_) was added. The absorbance of the chromophore formed was measured using a spectrophotometer (DU640i, Beckman) at 546 nm. All tests were performed in triplicate. Percent inhibition of the nitric oxide generated was measured by comparing the absorbance values of control and test preparations (equation 1). Curcumin was used as a reference material.

### Superoxide anion radical scavenging activity

The method described by Chang *et al.*[15] was used to investigate the superoxide anion scavenging activity of different fractions obtained from PEPN. The reaction mixture was prepared by dissolving Na_2_ CO_3_ (0.53 gm), EDTA (0.004 gm) and xanthine in 100 ml of distilled water. Ten ml of NBT solution (0.025 mM) was added to the reaction mixture. From this solution, 995 *µ*l with xanthine was further added to 5*µ*l of each fraction of PEPN and the reference compound, in different concentrations in distilled water. After 15 minutes, the absorbance was measured using a spectrophotometer (DU640i, Beckman) at 560 nm. The reaction mixture with xanthine oxidase was used as a control, whereas BHT was used as a reference compound. All tests were performed in triplicate. Percent inhibition was calculated by comparing the results of control and test samples (equation 1).

### Hydroxyl radical scavenging activity

Hydroxyl radical scavenging activity was measured comparing deoxyribose and PEPN for hydroxyl radical generated by the Fe^3+^-ascorbate-EDTA-H_2_ O_2_ system (Fenton reaction), using the method of Kunchandy and Rao.[16] The reaction mixture containing (1.0 ml) 100 *µ*l 2-deoxy-2-ribose (28 mM in 20 mM phosphate buffer, pH 7.4), 500 *µ*l of each fraction of PEPN and the reference compound in phosphate buffer (20 mM, pH 7.4), 200 *µ*l 1.04 mM EDTA, and 200 *µ*M FeCl_3_ (1:1 v/v), 100 *µ*l 1.0 mM H_2_ O_2_ and 100 *µ*l 1.0 mM ascorbic acid, was incubated at 37°C for an hour. One ml of 1% thiobarbituric acid and 1.0 ml of 2.8% trichloroacetic acid were added and incubated at 100°C for 20 min. After cooling, absorbance was measured using a spectrophotometer (DU640i, Beckman) at 532 nm, against a control preparation containing deoxyribose and buffer. Catechin was used as a positive control. Reactions were carried out in triplicate. Percent inhibition was determined by comparing the results of the test and control samples (equation 1).

### Amount of total phenolic compounds

Total soluble phenolic compounds present in the different fractions of PEPN were determined with the Folin-Ciocalteu reagent, according to the method suggested by Slinkard and Singleton.[17] To 0.1 ml of each fraction of PEPN (1 mg/ml in distilled water) in Erlenmeyer flask, one ml Folin-Ciocalteu reagent was added. After three minutes, 3 ml 2% Na_2_ CO_3_ was added. Subsequently, the mixture was shaken for 2 h at room temperature and absorbance was measured using a spectrophotometer (DU640i, Beckman) at 760 nm. All tests were performed in triplicate. The concentration of total phenolic compounds in samples was determined as *µ*g pyrocatechol equivalents, using the following equation obtained from a standard pyrocatechol graph:

Absorbance=0.001×pyrocatechol(μg)+0.0033

### Statistical analysis

Data are mean ± SD of three measurements. Statistical analysis was performed by the Student t-test and by anova. *P* < 0.05 was regarded as significant.

## Results

### Total antioxidant activity

In the present study, the antioxidant activity of the different fractions (R1, R2 & R3) of PEPN was determined using the ammonium thiocyanate method. All the fractions showed antioxidant property [Table 1] in a concentration dependent manner. Percent inhibition in case of R3 was more than R2. The fraction R1 showed minimum percent inhibition. All the fractions showed less percent inhibition than reference (α-tocopherol). The results indicate that the fractions R2 and R3 of PEPN significantly (*P* < 0.05) inhibited linoleic acid peroxidation.

**Table 1:** Inhibition (%) of lipid peroxidation in a linoleic acid emulsion by different fractions (R1, R2 and R3) of pet ether extract of *Piper nigrum* (PEPN) and by α-tocopherol (α -Tph)

*Quantity of the fraction used in *µ*g*	*% of Inhibition*			
	
	*R1*	*R2*	*R3*	*α-Tph*
50	4.33±5.25*	47.12±5.24*	49.94±5.78*	60.36±4.35*
100	7.58±2.80	50.56±1.32*	52.59±4.85*	65.32±5.48*
250	8.18±6.50	54.97±2.86	56.19±5.22*	68.44±2.57*
500	10.21±3.95	58.89±2.51*	60.48±3.33*	76.47±5.12*

Data are means ± SD of three measurements.

* *P* < 0.05 compared to control

### DPPH free radical scavenging activity

The DPPH radical is considered to be a model of lipophilic radical. A chain reaction in lipophilic radicals was initiated by lipid autoxidation. The scavenging effects of different fractions of PEPN and BHA on the DPPH radical are illustrated in Table 2. R2 and R3 had significant scavenging effects on the DPPH radical, which increased with increasing concentration from 50-250 *µ*g/ml. The scavenging effect of all the fractions was lower than that of BHA. The fraction R1 showed negligible percent inhibition. The fraction R2 and R3 possess statistically significant DPPH free radical scavenging activity (*P* < 0.05).

**Table 2:** 1, 1-diphenyl-2-picryl-hydrazol (DPPH) free radical scavenging activity of different fractions (R1, R2 and R3) of pet ether extract of *Piper nigrum* (PEPN) and butylated hydroxy anisole

*Quantity of the fraction used in *µ*g*	*% of Inhibition*			
	
	*R1*	*R2*	*R3*	*BHA*
50	3.56±1.32	12.58±2.8*	12.32±5.48*	38.59±4.85*
100	5.89±2.51	38.21±3.95*	38.47±5.12*	62.48±3.33*
150	8.97±2.86	43.18±6.55*	43.44±2.57*	70.19±5.22*
200	10.12±5.24	52.33±5.25*	52.36±4.35*	78.94±5.78*
250	12.41±6.62	61.24±7.58	61.11±4.98*	82.59±4.26*

Data are means ± SD of three measurements

* *P* < 0.05 compared to control

### Nitric oxide radical scavenging activity

The percent inhibition of nitric oxide generation by different fractions of PEPN is shown in Table 3. The antioxidant activity of all the three fractions was less than curcumin, which was used as a reference compound. It was also observed that R3 inhibits the production of nitric oxide radicals more actively than R2 and R1.

**Table 3:** Inhibition % of nitric oxide radicals by different fractions (R1, R2 and R3) of pet ether extract of *Piper nigrum* (PEPN) and curcumin

*Quantity of the fraction used in *µ*g*	*% of Inhibition*			
	
	*R1*	*R2*	*R3*	*Curcumin*
20	5.23±3.28*	10.54±2.96*	28.54±3.98*	51.22±3.55
40	8.57±2.24*	21.23±4.58	42.29±5.88*	70.06±2.54*
60	12.45±3.39	35.68±5.18*	48.52±4.19	75.45±2.48*
80	15.56±4.17	38.95±4.28*	52.04±5.11*	80.87±5.02*
100	18.22±4.31*	40.23±7.47*	55.68±4.48*	84.27±4.12*

Data are means ± SD of three measurements

* *P* < 0.05 compared to control

### Superoxide anion radical scavenging activity

All the fractions of PEPN were found to possess scavenging effects on superoxide anions, in a concentration dependent manner [Table 4]. R3 in the dose of 100*µ*g/ml inhibited the production of superoxide anion radicals by 70.22±3.55%, showing strong superoxide radical scavenging activity. However, the activity was lesser than the BHT.

**Table 4:** Superoxide anion scavenging activity of different fractions (R1, R2 and R3) of pet ether extract of *Piper nigrum* (PEPN) and butylated hydroxy toluene

*Quantity of the fraction used in *µ*g*	*% of Inhibition*			
	
	*R1*	*R2*	*R3*	*BHT*
20	8.95±4.28	25.56±4.17*	48.87±5.02*	65.04±5.11*
40	11.23±7.47	46.22±4.31*	57.27±4.12*	45.68±4.48*
60	15.68±5.18*	57.45±3.39*	64.45±2.48*	78.52±4.19*
80	17.23±4.58*	60.57±2.24*	68.06±2.54*	80.29±5.88
100	18.54±2.96*	62.23±3.28*	70.22±3.55*	81.54±3.98*

Data are means ± SD of three measurements

* *P* < 0.05 compared to control

### Hydroxyl radical scavenging activity

Hydroxyl radical scavenging activity was measured by studying the competition between deoxyribose and test (R1, R2 and R3) compounds for hydroxyl radical generated by Ferric^3+^ ascorbate-EDTA-H_2_ O_2_ system (Fenton reaction). The R2 and R3 fraction of PEPN were capable of reducing DNA damage at all concentrations, whereas R1 showed negligible or no effect [Table 5]. Catechin used as a standard was more effective than the fractions of PEPN in inhibiting the oxidative DNA damage.

**Table 5:** Hydroxyl radical scavenging activity of different fractions (R1, R2 and R3) of pet ether extract of *Piper nigrum* (PEPN) and catechin on deoxyribose damage

*Quantity of the fraction used in *µ*g*	*% of Inhibition*			
	
	*R1*	*R2*	*R3*	*Catechin*
50	17.27±4.12	35.23±3.28*	44.68±4.48*	50.22±3.55*
100	19.54±3.98	45.23±7.47*	55.54±2.96*	65.22±4.31*
250	20.08±2.54*	56.29±5.88	59.57±2.24*	68.23±4.58*
500	21.45±2.48*	58.52±4.19*	62.45±3.39*	69.67±5.18*
1000	21.87±5.02*	61.04±5.11*	63.56±4.17*	70.95±4.28

Data are means ± SD of three measurements

* *P* < 0.05 compared to control

### Amount of total phenolic compounds

The fraction R1, R2 and R3 were found to have 25.23, 38.56 and 56.98 *µ*g pyrocatechol equivalents of phenols respectively. The phenolic compounds may contribute directly to the antioxidative action.[18]

All the fractions showed free radical scavenging activity in different models in the present study. On comparison, it was found that fraction R3 has the highest antioxidant activity, whereas R1 has the least free radical scavenging activity. The antioxidant activity of these fractions might be due to inactivation of free radical or complex formation with metal ion, or a combination of both.

Most of the mammals have an inherent mechanism to prevent and neutralize the free radical induced damage. In biochemical system, superoxide radical and H_2_O_2_ react together to form a singlet oxygen and hydroxyl radical, which can attack and destroy almost all known biochemicals.[19] The hydroxyl radical produced may cause sugar fragmentation, base loss and leakage of DNA strand.[20] Hydroxyl radicals are the major ROS, causing lipid oxidation and enormous biological damage.[21] It is apparent from the present study that the PEPN not only scavenges off the free radicals but also inhibits the generation of free radicals. It was already reported that naturally occurring phenolic compounds have free radical scavenging properties, due to their hydroxyl groups.[22] Further, phenolic compounds are effective hydrogen donors, which make them antioxidant.[23]

It may be concluded that fractions obtained from pet ether extract of *P. nigrum* have significant antioxidant activity. The antioxidant potential may be attributed to the presence of polyphenolic compounds. These results are encouraging enough to pursue characterization of these fractions.
